# Probable Toxic Cause for Suspected Lychee-Linked Viral Encephalitis

**DOI:** 10.3201/eid2105.141650

**Published:** 2015-05

**Authors:** Peter S. Spencer, Valerie S. Palmer, Rajarshi Mazumder

**Affiliations:** Oregon Health & Science University, Portland, Oregon, USA (P.S. Spencer, V.S. Palmer);; Legacy Health, Portland (R. Mazumder)

**Keywords:** encephalitis, lychee fruit, litchi fruit, ackee, toxin

**To the Editor:** Paireau et al. (*1*) reported a spatiotemporal association between unexplained outbreaks of suspected acute encephalitis in children in northern Vietnam and the harvesting of lychee (litchi) fruit. The clinical, biologic, and immunologic characteristics of the patients suggested a viral etiology (*1*). However, the lychee-associated acute brain disorder, which has also been reported in Bangladesh and India (Bihar and West Bengal), could also result from ingestion of phytotoxins present in lychee fruit, specifically α-(methylenecyclopropyl)glycine (*2*), the lower homologue of the neurotoxic L-amino acid hypoglycine (*3**,**4*).

As previously described (*5*), ingestion of the hypoglycine-rich fruit of ackee, a relative of lychee, can induce a dose-dependent toxic hypoglycemic encephalopathy in poorly nourished children. The syndrome is best known from Jamaica, where ackee is widely eaten, and occurs most frequently in 2- to 10-year-old children, who develop severe hypoglycemia and metabolic acidosis. Clinical manifestations of Jamaican vomiting sickness include headache, thirst, sweating, vomiting, lethargy, seizures, coma, and death over a span of hours to days. Patients may be mildly to moderately febrile, and emesis may not be present in all cases. Heavy ingestion of the immature aril of ackee (*Blighia sapida*) or other members of the soapberry family (Sapindaceae), including lychee (*Litchi sinensis*), rambutan (*Nephelium lappaceum*), and longan (*Dimocarpus longan*), by an undernourished child with low glycogen/glucose stores probably has the potential to result in toxic hypoglycemic syndrome.

Assessment of finger-prick blood glucose levels, which may be markedly depressed in children with severe Sapindaceae fruit poisoning, provides a rapid and convenient screening tool to identify suspected cases. Intravenous administration of glucose is the first line of treatment, along with serial monitoring of glucose, serum aminotransferase, and serum creatinine levels. Restoration of body fluid, electrolytes, glucose, and pH balance is the goal of supportive treatment.

**Note added in proof.** Subsequent to the submission of this letter, a description was published of recent outbreaks of unexplained acute hypoglycemic encephalopathy in young children in Muzaffarpur, Bihar, coinciding with local lychee harvests (*6*).
